# Mayflies, stoneflies, and caddisflies of streams and marshes of Indiana Dunes National Lakeshore, USA

**DOI:** 10.3897/zookeys.556.6725

**Published:** 2016-01-21

**Authors:** R. Edward DeWalt, Eric J. South, Desiree R. Robertson, Joy E. Marburger, Wendy W. Smith, Victoria Brinson

**Affiliations:** 1 University of Illinois, Prairie Research Institute, Illinois Natural History Survey, 1816 S Oak St., Champaign, IL 61820; 2 University of Illinois at Urbana-Champaign, Department of Entomology, 320 Morrill Hall, 505 S. Goodwin Ave, Urbana, IL 61801; 3 Field Museum of Natural History, 1400 South Lake Shore Drive, Chicago, Illinois 60605; 4 Great Lakes Research and Education Center, Indiana Dunes National Lakeshore, 1100 N. Mineral Springs Road, Porter, Indiana 46304; 5 1545 Senator Lane, Ford heights, Illinois 60411

**Keywords:** Indiana Dunes National Lakeshore, Indiana Dunes State Park, Ephemeroptera, Plecoptera, Trichoptera, inventory

## Abstract

United States National Parks have protected natural communities for one hundred years. Indiana Dunes National Lakeshore (INDU) is a park unit along the southern boundary of Lake Michigan in Indiana, USA. An inventory of 19 sites, consisting of a seep, 12 streams, four marshes, a bog, and a fen were examined for mayflies (Ephemeroptera), stoneflies (Plecoptera), and caddisflies (Trichoptera) (EPT taxa). Volunteers and authors collect 35 ultraviolet light traps during summer 2013 and supplementary benthic and adult sampling added species not attracted by lights or that were only present in colder months. Seventy-eight EPT species were recovered: 12 mayflies, two stoneflies, and 64 caddisflies. The EPT richness found at INDU was a low proportion of the number of species known from Indiana: caddisflies contributed only 32.7% of known state fauna, mayflies and stoneflies contributed 8.4% and 2.3%, respectively. Site EPT richness ranged from one for a seep to 34 for an 8 m-wide stream. Richness in streams generally increased with stream size. Seven new state records and rare species are reported. The number of EPT species at INDU is slightly larger than that found at Isle Royale National Park in 2013, and the community composition and evenness between orders were different.

## Introduction

Extinction rates of North America freshwater fauna are 4–5 times higher than in terrestrial species and this trend is predicted to continue well into the future (Ricciardi and Rasmussen 1999). Master et al. (2000) suggest that aquatic invertebrates in the United States are highly imperiled, with mussels (Unionidae), crayfish (Decapoda: mostly Cambaridae), and stoneflies (Plecoptera) being rated as the top three most imperiled freshwater groups. Little is known of the original distribution, biology, and conservation status of most freshwater invertebrate species because they have been so poorly studied (Strayer 2006). Unfortunately, scientists are running out of high quality systems in which to study aquatic invertebrates due to the rapid degradation of their habitat. Large public properties such as United States National Parks may provide the minimally impacted aquatic habitat in which to study the biology of these once more widespread species. Inventory work within these parks may also shed light on the ability of public properties to support a portion of the regional species pool. The United States National Park System will celebrate its 100^th^ anniversary in 2016. This paper is a small tribute to the foresight of the United States government for its willingness to protect unique natural communities across the county.


**Figure 1.**
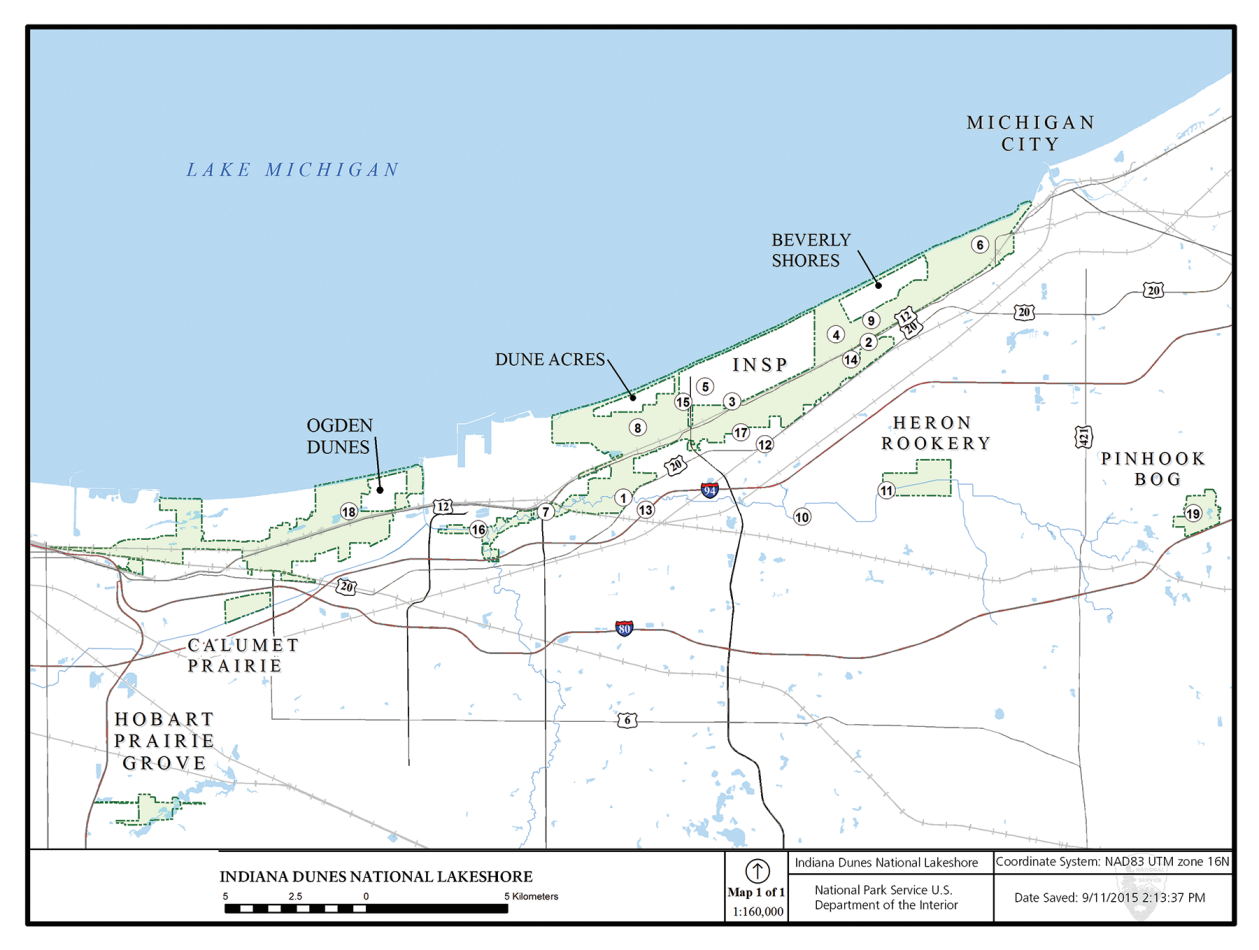
Sampling locations and extent of Indiana Dunes National Lakeshore and Indiana Dunes State Park (INSP). Site numbers in circles are from Table 1.

 Indiana Dunes National Lakeshore (INDU) is a unit of the United States National Park Service located in northwestern Indiana along the southern Lake Michigan shoreline. A mosaic of public and private property, it extends 24 km from Gary east to Michigan City (Fig. 1). The USA Congress authorized the park in 1966 after a half century of activism to preserve the unique physical features and associated vegetation (National Park Service 2015). Scientists know Indiana Dunes as the “birthplace of ecology” due to Cowles’ (1899) pioneering efforts on vegetative succession.

**Table 1.** Locations for sampling of Ephemeroptera, Plecoptera, and Trichoptera at Indiana Dunes National Lakeshore (INDU) and Indiana Dunes State Park (INSP) during 2013 and 2014. Waterbody type or stream wetted width (m) provided.

SiteID	County	Stream		Locality	Latitude	Longitude	Width (m)
1	Porter	East Arm L. Calumet R.	INDU	at Howe Rd.	41.62145	-87.09267	29
2	Porter	Trib. Beverly Rd. Marsh	INDU	at US-12	41.67135	-86.98812	2
3	Porter	Munson Ditch	INSP	9.3 km NE Crocker	41.65613	-87.05671	8
4	Porter	Beverly Dr. Marsh	INDU	9.7 km WSW Michigan City	41.67375	-87.00207	Marsh
5	Porter	Dunes Creek	INSP	9.4 km NE Crocker	41.65706	-87.05788	5
6	Porter	Kintzele Ditch	INDU	3.8 km W Michigan City	41.70249	-86.94065	8
7	Porter	East Arm L. Calumet R.	INDU	3.2 km N Crocker at IN-149	41.61701	-87.12574	29
8	Porter	Cowles Bog	INDU	6.9 km NNE Crocker	41.64396	-87.08664	Fen
9	Porter	Grand Marsh	INDU	E Broadway Ave. at boardwalk	41.67825	-86.98707	Marsh
10	Porter	East Arm L. Calumet R.	INDU	9.3 km ENE Crocker at Brummit Rd.	41.61544	-87.01653	25
11	Porter	East Arm L. Calumet R.	INDU	12.5 km ENE Crocker-Heron Rockery	41.62388	-86.98045	25
12	Porter	Munson Ditch	INDU	9.0 km NE Crocker at Hawleywood Rd.	41.64243	-87.04272	2
13	Porter	Marsh	INDU	Howe Rd.	41.62147	-87.09356	Marsh
14	Porter	Trib. Great Marsh	INDU	US-12 & CR 375E	41.66796	-86.99571	2
15	Porter	Dunes Creek	INDU	8.5 km NNE Crocker at Waverly Rd.	41.65221	-87.06731	5
16	Porter	East Arm L. Calumet R.	INDU	3.4 km ESE Ogden Dunes	41.61137	-87.15446	29
17	Porter	Seep Munson Ditch	INDU	9.0 km NE Crocker	41.64246	-87.04259	Seep
18	Porter	Long Lake	INDU	1.9 km WSW Ogden Dunes at Beach Rd.	41.61692	-87.20969	Marsh
19	La Porte	Pinhook Bog	INDU	6.1 km S Waterford at N. Wozniak Rd.	41.61641	-86.84982	Bog

The Wisconsinan ice sheets receded approximately 10,000 years ago leaving vast deposits of sand that formed the Lake Michigan shoreline. Changing lake levels gave rise to a series of beachfronts, sand dunes, and interdunal swales. Moraines serve as drainage divides that form several streams that flow to Lake Michigan through INDU (Hill 1974). These streams and marshes of INDU provide habitat for a wide range of plants and animals. Interdunal swales are extensive and open, or partly wooded. Several small streams, including Dunes Creek, Kintzele Ditch, and Munson Ditch, enter the swales then reform channels to exit via Lake Michigan.

The largest flowing water resource in INDU, the East Arm of the Little Calumet River, flows from east to west, beginning midway along the Porter and La Porte county line and emptying into Lake Michigan near Ogden Dunes. Most of the river’s drainage is not contained within INDU, although the most sinuous and heavily wooded stretches are contained within park boundaries. Much of this highly modified system was channelized early in the 20^th^ century to hasten drainage. Water quality of the East Arm of the Little Calumet River is moderately impaired and advisories against fish consumption related to mercury and PCB contamination and contact due to pathogens have been posted (Lake Michigan Coastal Program 2015). Still, sections of the river upstream of the most industrialized area flow naturally and harbor remnants of the fish and macroinvertebrate communities that have always been present in streams of the region. Near the western end of INDU is Cowles Bog, a fen surrounded by marshland. To the far east is Pinhook Bog, a true acidic bog, supporting a bog plant community.

While vertebrate species abundance and community structure are generally well known for many National Park units, information on the invertebrate assemblages is often lacking. Ephemeroptera (mayflies), Plecoptera (stoneflies), and Trichoptera (caddisflies) (EPT species) are environmentally sensitive aquatic insects that are routinely used in monitoring of water quality (Barbour et al. 1999). Their taxonomy and distribution are relatively well known in the Midwest (Burks 1935, DeWalt et al. 2005, DeWalt et al. 2012, DeWalt and Grubbs 2011, Frison 1935, Grubbs et al. 2012, Houghton 2012, Randolph and McCafferty 1998, Ross 1944, Waltz and McCafferty 1983). This makes EPT an appropriate target for inventories within INDU.

The objectives of this study are to conduct an inventory of the EPT present in INDU, asking the following questions of the resulting data:

1. What is the species richness of EPT and the distribution of species within orders and families within the study area?
2. How does INDU EPT richness compare to known richness of EPT in Indiana?
3. Are there trends in EPT richness versus waterbody type and stream wetted width?
4. Are there any species of conservation significance inhabiting INDU?

This project is the second of four studies on the EPT of upper Great Lakes National Parks. A comparison to the results of inventory work on Isle Royale National Park, Michigan is discussed (DeWalt and South 2015).

## Methods

Sampling of EPT taxa was greatly facilitated by a dedicated group of volunteers, organized by JEM and WWS, who set up and retrieved UV light traps from various locations in INDU and Indiana Dunes State Park (IDSP). The two locations in IDSP are immediately adjacent to INDU and will from here forward be referred to as INDU sites. Light trap units consisted of a portable camping light modified with a UV spectrum fluorescent bulb, a large white plastic tray, a 250 ml Nalgene ™ bottle, forceps, and a supply of 95% EtOH. Several such units were provided to INDU for volunteer use. Volunteers placed traps in an inconspicuous location near streams or marshes just before dark, often left them unattended, and then reclaimed them after approximately 1.5 hr. The contents of the tray were decanted into a fully labeled sample bottle and returned to park headquarters. Often, more than one waterbody was trapped per night.

Ultraviolet light traps are an efficient means of sampling caddisfly adults. However, mayflies and stoneflies required supplementary sampling in stream sites to collect species that do not come to lights or that emerged as adults in colder times of the year. These sites were sampled with dipnets, beating sheets, and sweepnets on several occasions in May, 2013 and early April, 2014 (Table 2).

**Table 2.** Dates or ranges of dates of sampling events and types of sampling devices used to collect Ephemeroptera, Plecoptera, and Trichoptera in Indiana Dunes National Lakeshore and Indiana Dunes State Park. Date format is month/day/year.

	Sample Dates											
SiteID	5/4/2013	6/27/2013	7/1/2013	7/10-11/2013	7/16-17/2013	7/22/2013	7/31-8/1/2013	8/8-9/2013	8/27/2013	4/6/2014	10/21/2014	Events
1	Dipnet			Dipnet, Sweep, UV			UV		UV	Dipnet	UV	7
2					UV		UV			Dipnet		3
3			UV		UV		UV					3
4		UV			UV							2
5					UV		UV					2
6					UV		UV					2
7									UV			1
8		UV					UV					2
9		UV					UV					2
10				UV				UV				2
11					UV			UV		Dipnet		3
12				UV			UV					2
13			UV				UV			Dipnet		3
14					UV		UV			Dipnet		3
15				Sweep, UV			UV					3
16				UV					UV			2
17				Handpicking								1
18						UV						1
19					UV			UV				2
											Total	46

Sample sorting was also volunteer facilitated with INDU managers, local high school students, and authors attending a two-day sample sorting workshop at INDU headquarters on October 16-17, 2013. Under supervision of the authors, volunteers sorted EPT by order and body size into separate vials of 95% EtOH. Samples were returned to the Illinois Natural History Survey (INHS) for additional sorting and labeling. Identification was to species where possible. Nomenclature followed that of Mayfly Central (2015), Plecoptera Species File (DeWalt et al. 2015), and the Trichoptera World Checklist (Morse 2015).

All specimens have been accessioned into the INHS Insect Collection (INHS-IC). The INHS provides global access to specimen data through the INHS-IC database portal (http://inhsinsectcollection.speciesfile.org/InsectCollection.aspx). These data are also shared with the Global Biodiversity Information Facility. Raw specimen data are provided as a supplementary comma delimited file (Suppl. material 1).

To answer question one, EPT richness was compiled across all samples at a site and the number of species in each order and family tallied. Comparison of INDU EPT to published Indiana records was conducted using Randolph and McCafferty (1998) for mayflies, DeWalt and Grubbs (2011) for stoneflies, and Waltz and McCafferty (1983) and Rasmussen and Morse (2014) for caddisflies. The low richness for mayflies and stoneflies necessitated comparison by text alone, but for caddisflies a graphical comparison was possible.

Trends in species richness with stream wetted width (width of water at base flow) were investigated using Spearman Rank Correlation (Lowry 2015). Stream wetted width was estimated at each site from comparison with road widths on satellite images viewed on Acme Mapper 2.1 (http://mapper.acme.com) and recorded to the nearest meter. In addition, wetted width was expressed as three stream size categories (1–2 m, 3–10 m, and 11–30) so that EPT richness mean ± SE could be graphically compared. The average richness of seeps, marshes, a bog, and a fen were similarly compared. Conservation significance was discussed based on species being new state records or having been rarely reported from Indiana.

## Results

Volunteers and authors collected 46 samples for an average of 2.4 visits/location at 19 locations (Table 1, Table 2).

**Figure 2.**
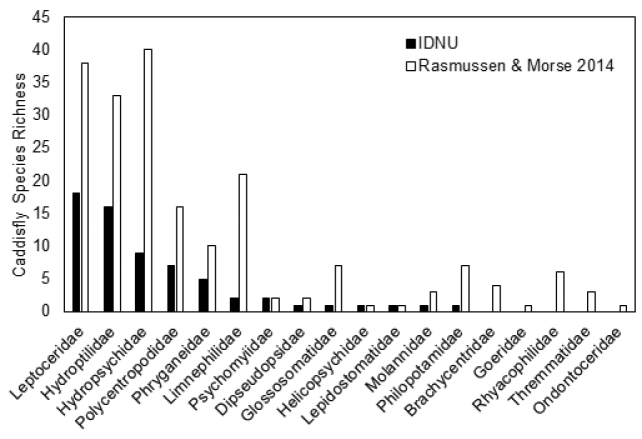
Comparison of caddisfly species richness within families at Indiana Dunes National Lakeshore versus Indiana records published by Rasmussen and Morse (2014).

*What is the species richness of EPT and the distribution of species within orders and families within the study area*? A total of 7,321 specimens were collected, resulting in 78 EPT species (Table 3). Mayflies contributed 12 species, most of which were in the families Baetidae (small minnow mayflies, five species) and Heptageniidae (flat-headed mayflies, five species). Stoneflies contributed only two species, one in the Perlidae (summer stone) and one in the Taeniopterygidae (willowfly, a winter-emerging stonefly). Caddisflies dominated EPT species richness with 64 species among13 families (Fig. 2). Four families contributed 78% of all caddisfly species: Leptoceridae (long-horned caddisflies, 18 species), Hydroptilidae (microcaddisflies, 16 species), Hydropsychidae (net-spinning caddisflies, nine species), and Polycentropodidae (finger-net caddisflies, seven species).

**Table 3.** Species of Ephemeroptera, Plecoptera, and Trichoptera collected from Indiana Dunes National Lakeshore and Indiana Dunes State Park during 2013 and 2014, Porter and La Porte counties, Indiana. *Indicates new state record.

	Sampling Stations-See Table 1																		
Taxon and Authority	1	2	3	4	5	6	7	8	9	10	11	12	13	14	15	16	17	18	19
**Ephemeroptera-mayflies**																			
**Baetidae**																			
*Baetis flavistriga* McDunnough	0	0	0	0	0	1	0	0	0	0	0	0	0	0	0	0	0	0	0
*Baetis intercalaris* McDunnough	23	0	0	0	0	0	0	0	0	0	0	0	0	0	0	0	0	0	0
*Callibaetis ferrugineus* (Walsh)	0	0	0	1	0	0	0	0	0	0	0	0	0	0	0	0	0	0	0
*Callibaetis fluctuans* (Walsh)	0	0	1	0	0	0	0	1	10	0	0	0	0	0	0	0	0	0	0
*Callibaetis pallidus* Banks*	0	0	0	2	0	0	0	0	0	0	0	0	0	0	0	0	0	0	0
**Caenidae**																			
*Caenis amica* Hagen	0	0	1	17	0	0	0	0	10	0	0	0	0	0	0	0	0	0	0
**Ephemeridae**																			
*Hexagenia limbata* (Serville)	8	0	0	0	0	0	2	0	0	2	0	0	0	0	0	0	0	0	0
**Heptageniidae**																			
*Heptagenia elegantula* (Eaton)	1	0	0	0	0	0	0	0	0	0	0	0	0	0	0	0	0	0	0
*Maccaffertium exiguum* (Traver)	3	0	0	0	0	0	0	0	0	0	0	0	0	0	0	0	0	0	0
*Maccaffertium terminatum* (Walsh)	112	0	0	0	0	0	2	0	0	92	101	0	0	0	0	4	0	0	0
*Maccaffertium vicarium* (Walker)	0	0	0	0	0	0	0	0	0	0	1	0	0	0	0	0	0	0	0
*Stenacron interpunctatum* (Say)	12		23		119	23	1	0	0	2	9	0	0	0	0	0	0	0	0
**Plecoptera-stoneflies**																			
**Perlidae**																			
*Perlesta lagoi* Stark	0	0	25	0	28	5	0	0	0	3	8	0	0	0	1	2	0	0	0
**Taeniopterygidae**																			
*Taeniopteryx burksi* Ricker & Ross	0	0	0	0	0	0	0	0	0	0	1	0	0	0	0	0	0	0	0
**Trichoptera-caddisflies**																			
**Dipseudopsidae**																			
*Phylocentropus placidus* (Banks)	0	0	5	0	7	0	0	0	0	0	0	0	0	0	0	0	0	0	0
**Glossosomatidae**																			
*Protoptila maculata* (Hagen)	0	0	0	0	0	0	0	0	0	0	0	0	0	0	0	1	0	0	0
*Protoptila* sp.	0	0	0	0	0	0	0	0	0	0	0	0	0	0	0	2	0	0	0
**Hydropsychidae**																			
*Cheumatopsyche analis* (Banks)	13	10	76	7	29	16	0	1	0	19	167	28	1	0	0	10	0	0	4
*Cheumatopsyche campyla* Ross	0	0	0	0	0	0	0	0	0	0	0	0	0	0	0	0	0	6	0
*Cheumatopsyche oxa* Ross	0	0	2	0	0	0	0	0	0	1	0	0	0	0	0	1	0	0	0
*Cheumatopsyche pasella* Ross	0	0	0	1	0	0	0	0	0	0	0	0	0	0	0	0	0	0	0
*Cheumatopsyche* sp.	150	0	13	0	0	0	0	5	0	60	0	0	1	2	5	8	0	0	1
*Hydropsyche betteni* Ross	81	3	96	8	12	45	24	0	0	10	254	6	1	20	2	93	0	0	3
*Hydropsyche bronta* Ross	14	2	13	0	0	1	187	0	0	0	4	0	0	1	0	3	0	0	0
*Hydropsyche morosa* group	3	0	0	0	0	0	0	0	0	0	0	0	0	0	0	0	0	0	0
*Hydropsyche simulans* Ross	0	1	0	0	0	0	0	0	0	0	0	0	0	0	0	0	0	1	2
*Hydropsyche sparna* Ross	2	2	7	1	2	1	0	0	0	4	111	0	0	1	0	5	0	0	2
*Hydropsyche* sp.	0	0	1	0	217	0	300	2	0	28	34	0	20	3	0	5	0	0	3
*Potamyia flava* (Hagen)	0	0	0	0	0	0	14	0	0	0	0	0	0	0	0	1	0	0	0
**Hydroptilidae**																			
*Agraylea multipunctata* Curtis	0	0	0	0	0	0	0	2	1	0	0	0	0	0	0	0	0	1	0
*Hydroptila ajax* Ross	0	0	0	0	1	0	0	0	0	1	0	0	0	0	0	4	0	0	0
*Hydroptila albicornis* Hagen	0	0	1	0	0	0	0	0	0	0	0	0	0	0	0	0	0	0	0
*Hydroptila angusta* Ross	1	0	16	3	0	1	0	0	0	0	0	0	0	0	0	0	0	0	1
*Hydroptila armata* Ross	0	0	29	8	1	3	12	0	0	3	0	0	0	0	0	2	0	0	0
*Hydroptila consimilis* Morton	3	0	7	0	14	32	44	0	0	1	8	0	0	0	1	100	0	1	1
*Hydroptila grandiosa* Ross	0	0	0	0	0	0	3	0	0	0	1	0	0	0	0	1	0	0	0
*Hydroptila perdita* Morton	0	0	0	1	0	0	3	0	0	0	0	0	0	0	0	0	0	0	0
*Hydroptila spatulata* Morton	0	0	0	0	0	0	2	0	0	0	0	0	0	1	0	0	0	0	0
*Hydroptila waubesiana* Betten	57	7	26	24	13	18	13	3	1	12	1	0	1	0	14	27	0	7	1
*Hydroptila* sp.	0	1	22	0	38	2	275	1	19	78	12	0	0	2	0	94	0	16	13
*Ochrotrichia* sp.	0	0	0	0	0	0	1	0	0	0	0	0	0	0	0	0	0	0	0
*Orthotrichia aegerfasciella* (Chambers)	0	0	3	8	0	3	0	9	0	0	7	0	0	0	0	1	0	4	0
*Orthotrichia cristata* Morton	0	0	1	28	0	0	0	0	0	0	2	0	0	0	0	0	0	1	5
*Orthotrichia* sp.	0	0	0	0	0	0	2	0	9	0	0	0	0	0	0	0	0	0	2
*Oxyethira forcipata* Mosely*	0	0	1	0	0	0	0	0	0	0	0	0	0	0	0	0	0	0	0
*Oxyethira pallida* (Banks)	2	0	51	366	57	55	41	0	64	0	29	0	1	0	3	41	0	306	71
*Oxyethira serrata* Ross	0	0	0	5	0	0	0	0	0	0	0	0	0	0	0	0	0	0	0
*Oxyethira* sp.	0	0	0	0	0	0	0	0	19	0	0	0	0	0	0	0	0	0	0
**Lepidostomatidae**																			
*Lepidostoma* sp.	0	0	0	0	0	0	0	0	0	0	0	0	0	1	0	0	1	0	0
**Leptoceridae**																			
*Ceraclea alagma* (Ross)	0	0	0	0	0	0	0	3	2	0	0	0	0	0	0	0	0	0	1
*Ceraclea punctata* (Banks)*	0	0	0	0	0	0	0	0	0	0	1	0	0	0	1	36	0	0	0
*Ceraclea tarsipunctata* (Vorhies)	0	0	0	8	0	0	0	0	0	0	0	0	0	0	0	0	0	0	0
*Ceraclea* sp.	0	0	2	0	0	0	0	0	0	0	0	0	0	0	0	0	0	3	0
*Leptocerus americanus* (Banks)	0	0	1	62	0	1	0	247	125	0	2	0	0	0	0	3	0	8	7
*Nectopsyche diarina* (Ross)	0	0	0	0	0	0	0	0	0	0	0	0	0	0	0	1	0	0	0
*Nectopsyche exquisita* (Walker)	0	0	0	0	0	0	3	0	0	0	0	0	0	0	0	6	0	1	0
*Nectopsyche pavida* (Hagen)	0	0	0	1	0	0	0	0	0	0	0	0	0	0	0	0	0	0	0
*Nectopsyche* sp.	0	0	0	3	0	1	0	0	1	0	0	0	0	1	0	7	0	0	1
*Oecetis cinerascens* (Hagen)	0	3	1	12	0	2	0	0	2	1	5	0	1	1	0	5	0	186	1
*Oecetis ditissa* Ross	0	0	0	2	0	1	0	0	0	0	0	0	0	0	0	0	0	0	0
*Oecetis inconspicua* (Walker)	2	1	15	52	6	21	12	6	13	5	27	0	1	1	2	15	0	75	25
*Oecetis* n.sp.	0	0	0	0	0	2	0	0	0	0	0	0	0	0	0	0	0	0	0
*Oecetis nocturna* Ross	0	0	0	0	0	1	0	0	0	0	0	0	0	0	0	0	0	0	0
*Oecetis ochracea* (Curtis)*	0	0	0	0	0	1	0	0	0	0	0	0	0	0	0	0	0	0	0
*Oecetis persimilis* (Banks)	0	0	0	0	0	0	0	0	0	1	0	0	0	0	0	0	0	0	0
*Oecetis* sp.	0	0	0	0	0	0	0	1	1	0	0	0	0	0	0	0	0	0	0
*Triaenodes aba* Milne	1	0	1	270	0	0	0	145	12	0	0	0	0	0	0	0	0	3	1
*Triaenodes melacus* Ross	0	0	11	1	19	3	0	0	0	0	1	0	0	0	0	3	0	0	0
*Triaenodes nox* Ross	0	0	1	0	0	0	0	0	0	0	0	0	0	0	0	77	0	0	0
*Triaenodes tardus* Milne	0	0	2	42	0	1	0	3	17	8	1	0	0	0	0	0	0	79	5
*Triaenodes* sp.	0	0	0	0	0	0	0	0	0	0	0	0	0	0	0	0	0	0	1
**Limnephilidae**																			
*Platycentropus radiatus* (Say)	0	2	1	1	0	0	0	0	1	0	0	0	1	0	0	0	0	0	0
*Pycnopsyche guttifera* (Walker)	1	0	0	0	0	0	0	0	0	0	0	0	0	0	0	0	0	0	0
*Pycnopsyche* sp.	6	0	0	0	0	0	0	0	0	0	0	0	0	1	0	0	0	0	0
**Molannidae**																			
*Molanna tryphena* Betten*	0	0	1	0	2	0	0	0	0	0	1	0	0	0	0	0	0	0	0
**Philopotamidae**																			
*Chimarra obscura* (Walker)	0	0	0	0	0	36	0	0	0	0	0	0	0	0	0	0	0	0	0
**Phryganeidae**																			
*Agrypnia vestita* (Walker)	0	0	0	0	0	0	0	0	0	0	0	0	0	0	0	2	0	0	0
*Banksiola crotchi* Banks	0	0	0	2	0	0	0	1	0	0	0	0	0	0	0	0	0	2	1
*Phryganea cinerea* Walker	0	0	0	0	0	0	0	0	0	0	3	0	0	0	0	0	0	0	1
*Ptilostomis ocellifera* (Walker)	0	12	3	0	5	0	0	0	0	1	0	0	0	3	0	1	0	0	0
*Ptilostomis postica* (Walker)	0	0	1	0	0	0	0	0	0	0	0	0	0	0	0	0	0	0	0
*Ptilostomis* sp.	1	2	0	0	0	0	0	0	0	0	0	0	0	0	0	0	0	0	0
**Polycentropodidae**																			
*Cernotina calcea* Ross*	0	0	0	0	0	0	0	0	0	0	0	0	0	0	0	3	0	0	0
*Neureclipsis crepuscularis* (Walker)	1	0	0	0	0	0	0	0	0	0	0	0	0	0	0	0	0	0	0
*Nyctiophylax moestus* Banks	17	0	0	1	0	0	0	0	0	8	61	0	0	0	0	5	0	0	0
*Plectrocnemia cinerea* (Hagen)	0	0	0	1	0	0	0	0	0	0	0	0	0	0	0	9	0	0	0
*Plectrocnemia clinei* Milne*	0	0	0	0	0	0	0	0	0	0	0	0	0	0	0	0	0	0	3
*Plectrocnemia crassicornis* (Walker)	0	0	1	0	0	0	0	0	0	0	0	0	0	0	0	0	0	0	0
*Polycentropus confusus* Hagen	0	0	0	0	0	0	0	0	0	1	3	0	0	0	0	0	0	0	0
*Polycentropus* sp.	1	0	1	0	0	0	0	0	0	27	0	0	0	0	0	9	0	1	0
**Psychomyiidae**																			
*Lype diversa* (Banks)	7	0	1	1	0	0	2	0	0	4	21	0	0	0	0	1	0	0	0
*Psychomyia flavida* Hagen	0	0	0	0	0	0	2	0	0	0	0	0	0	0	0	0	0	0	0
Total count	522	46	463	939	570	276	945	430	307	372	876	34	28	38	29	588	1	701	156
Ephemeroptera richness	6	0	3	2	1	2	3	1	2	3	3	0	0	0	0	1	0	0	0
Plecoptera richness	0	0	1	0	1	1	0	0	0	1	2	0	0	0	1	1	0	0	0
Trichoptera richness	16	10	30	26	13	21	16	11	12	16	21	2	7	11	7	29	1	16	19
Total EPT richness	22	10	34	28	15	24	19	12	14	20	26	2	7	11	8	31	1	16	19

*How does INDU EPT richness compare to known richness of EPT in Indiana*? In Indiana there are at least 143 species of mayflies in 16 families (Randolph and McCafferty 1998). Those found within INDU accounted for only 8.4% of the Indiana fauna. DeWalt and Grubbs (2011) reported 87 species of stoneflies within eight families in Indiana. INDU richness amounted to only 2.3% of the known Indiana fauna. Waltz and McCafferty (1983) listed 190 species of caddisflies from Indiana. Rasmussen and Morse (2014), in their compendium of Nearctic distributions, listed a total of 196 species in 18 families. Samples in INDU recovered only 32.7% of the Indiana caddisfly fauna reported by Rasmussen and Morse (2014) (Fig. 2).

**Figure 3.**
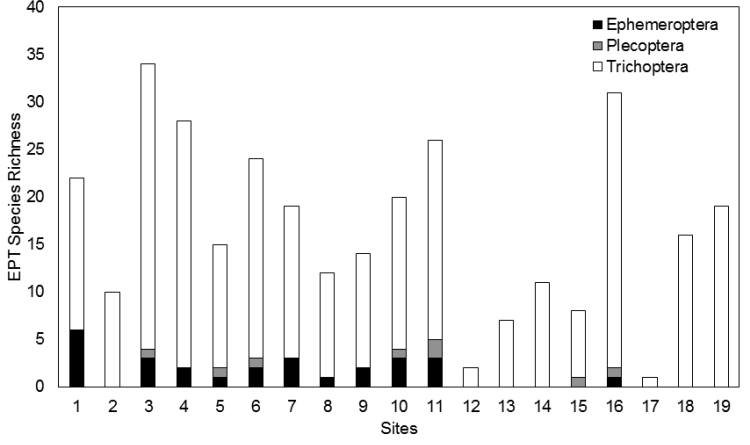
EPT richness found at each of 19 locations in Indiana Dunes National Lakeshore. Refer to Table 1 for specific site information.

**Figure 4.**
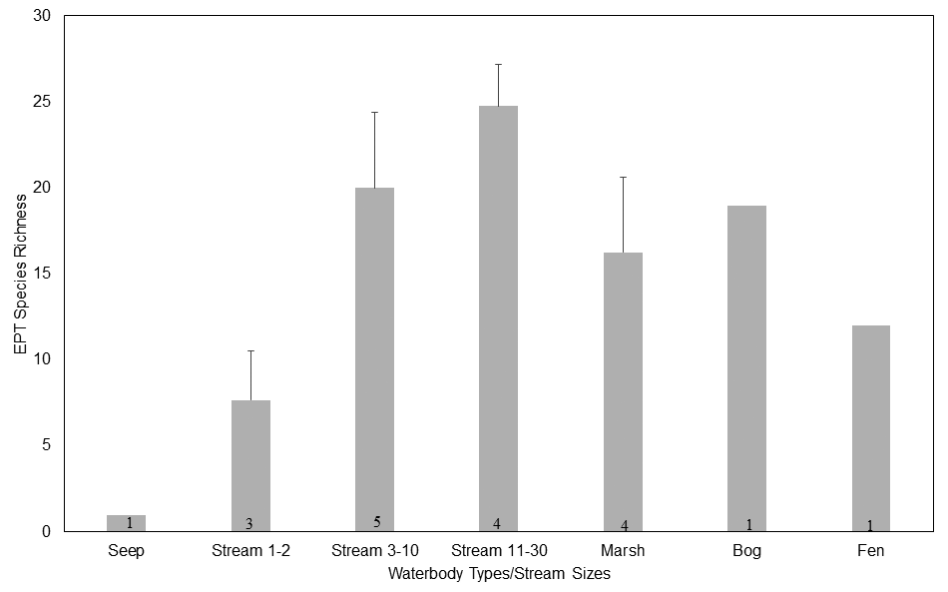
Mean ± SE of EPT richness by stream size and waterbody type within Indiana Dunes National Lakeshore and Indiana Dune State Park. Number in bar indicates sample size.

*Are there trends in EPT richness versus waterbody type and stream wetted width*? Richness of EPT varied greatly across stream sizes and water body types in INDU (Table 3, Fig. 3). At all sites caddisflies dominated richness. Among streams, EPT richness increased with wetted width (Fig. 4), but the correlation was not quite significant (R = 0.55, p = 0.06, df = 16). Small streams of less than 2 m wetted width rarely produced more than 10 species, while larger streams averaged 20 or more species. One seep, densely vegetated by skunk cabbage, *Symplocarpus foetidus* (L.) Salisb. ex Barton, produced only the empty, coarse sand cases of the caddisfly *Lepidostoma* sp. This population probably died during an extensive drought of the previous year, though their cases remained. Cowles Bog, actually a fen, produced one mayfly and 11 caddisfly species. Pinhook Bog, the only acid bog among the sampling sites, produced 19 caddisfly species. Marshes produced an average of 16.5 EPT species, 89% of species captured there being caddisflies. Marshes, the fen, and bog supported a similar caddisfly fauna, exhibiting little in the way of uniqueness.

*Are there any species of conservation significance inhabiting INDU*? We collected several rare species and seven that were new records for Indiana. In addition, one potentially undescribed species of caddisfly was collected. A discussion of these records follows.

Ephemeroptera

Baetidae – Small Minnow Mayflies

*Callibaetis pallidus* Banks. This is a new state record. Randolph and McCafferty (1998) did not report the species from Indiana. However, Check (1982), in an unpublished master’s thesis, listed Indiana as part of the distribution of the species. This is the first published record of the species in Indiana. Two females were taken from Beverly Dr. Marsh (Site 4).

Trichoptera

Polycentropodidae – Fingernet Caddisflies

*Cernotina calcea* Ross. This is a new state record. Ross (1938) described this species from the nearby Kankakee River, Illinois. It has not been reported from Indiana (Waltz and McCafferty 1983), Michigan (Leonard and Leonard 1949a, b), Minnesota (Houghton 2012), Ohio (Armitage et al. 2011), or Wisconsin (Longridge and Hilsenhoff 1973). One male and two females were collected from the East Arm of the Little Calumet River (Site 16).

*Plectrocnemia clinei* Milne. This is a new state record. Waltz and McCafferty (1983) did not list this species from Indiana. It has only been reported from Ohio (Armitage et al. 2011) and Minnesota (Houghton 2012) in the region. Two males and one female were taken from Pinhook Bog (Site 19).

*Plectrocnemia crassicornis* (Walker). This species has rarely been collected in Indiana. We collected a single specimen of the distinctive female from Munson Ditch (Site 3). The only published Indiana record is a single female from a nearby locality: INHS-Trichoptera-54964, “Michigan City, Ind. [La Porte Co.] Trail Creek June 21, 1957 John Lowe” (Waltz and McCafferty 1983). A second unpublished record exists in the INHS Insect Collection: INHS-Trichoptera-54963, “Morgan-Monroe St. Forest 7 mi. S. of Martinsville IND. May 16, 1962 H. H. Ross & J. Kingsolver at light”. It is the only male specimen that has been collected in the state. The species has been reported from all states in the region (Armitage and Hamilton 1990, Armitage et al. 2011, Houghton 2012, Leonard and Leonard 1949b, Longridge and Hilsenhoff 1973, Ross 1944).

*Polycentropus confusus* Hagen. This species, too, is rarely collected in Indiana, its only published record is from Jefferson County in Clifty Falls State Park (Waltz and McCafferty 1983). It is known from all other neighboring states except Illinois (Armitage and Hamilton 1990). Three males and one female were collected from two locations on the East Arm Little Calumet River (Sites 10 & 11).

Hydroptilidae – Microcaddisflies

*Hydroptila albicornis* Hagen. This is the first northern Indiana record. Waltz and McCafferty (1983) previously reported it from the town of Shoals along the East Fork of the White River. A single female specimen was taken along Munson Ditch (Site 3). The species is known from all neighboring states except Michigan and Kentucky (Blickle 1979).

*Oxyethira forcipata* Mosely. This new state record is represented by a single male collected from Munson Ditch (Site 3). The species is known from all neighboring states except Kentucky (Blickle 1979) and has been recently demonstrated to occur in high incidence across Ohio, especially in the Erie-Ontario Lake Plains and the Western Allegheny Plateau (Armitage et al. 2011).

*Oxyethira serrata* Ross. This species is rare in Indiana, its only other record being from Lake Maxinkuckee in Marshall County (Waltz and McCafferty 1983). Four females were collected from Beverly Dr. Marsh (Site 4). The species in known from neighboring Illinois and Michigan (Blickle 1979).

Molannidae – Hoodcase Case Caddisflies

*Molanna tryphena* Betten. This is a new state record. The species is represented by three males and one female from Munson Ditch and Dunes Creek in IDSP and in the East Arm of the Little Calumet River at Heron Rookery (Sites 3, 5, 11). The species is known from Michigan (Leonard and Leonard 1949b), Minnesota (Houghton 2012), and Wisconsin (Longridge and Hilsenhoff 1973) within the Midwest, Great Lakes region. This is now the fourth *Molanna* known from Indiana (Waltz and McCafferty 1983).

Leptoceridae – Longhorn Caddisflies

*Ceraclea punctata* (Banks). This is a new state record. Five males and 33 females were collected from two locations on the East Arm of the Little Calumet River and from Dunes Creek (Sites 11, 15, 16). The species is known regionally from Illinois (Ross 1944), Michigan (Leonard and Leonard 1949b), Ohio (Armitage et al. 2011), and Wisconsin (Longridge and Hilsenhoff 1973).

*Nectopsyche pavida* (Hagen). This species is rarely collected in Indiana and is known only from Harrison (far south) and LaGrange (northeast corner) counties (Waltz and McCafferty 1983). A single female was taken from Beverly Dr. Marsh (Site 4). Though this species has been rarely collected in surrounding states (Leonard and Leonard 1949b, Longridge and Hilsenhoff 1973, Ross 1944), recent work has provided 30 locality records scattered across Ohio (Armitage et al. 2011).

*Oecetis ochracea* (Curtis). This is tentatively a new state record. A single female from Kintzele Ditch (Site 6) was collected. In the region, it is known from Ohio (Armitage et al. 2011) and Wisconsin (Longridge and Hilsenhoff 1973).

*Oecetis inconspicua* (Walker) complex. One male and one female from Kintzele Ditch were recovered that superficially resemble *Oecetis inconspicua*. The male specimen displays an elongate and dorsally directed appendage at the base of the inferior appendage (clasper), whereas in *Oecetis inconspicua* figured by Ross (1944), this projection is small and squat. Some small differences are also apparent in the female. Description of this new species must be conducted as part of a revision of the complex, using both morphological characters and gene sequence data.

*Triaenodes aba* Milne. This species is known from Indiana by a single record from the Tippecanoe River in Kosciusko County (Waltz and McCafferty 1983). We collected over 400 females from the following sites: 1, 3, 4, 8, 9, 18, 19. Approximately 99.5% of these specimens were from marsh, bog, or fen habitat. It is likely to be abundant in such habitats elsewhere in Indiana, as has been the case in Ohio (Armitage et al. 2011). The species is known from all states that border Indiana (Glover 1996).

## Discussion

A total of 78 EPT species was recovered from samples within INDU and IDSP. Included among these were seven new state records consisting of one mayfly and six caddisflies. Additionally, there is the potential for one caddisfly species new to science in the *Oecetis inconspicua* complex (Floyd 1995, Zhou et al. 2010). While caddisflies were dominant in both numbers of individuals and species richness, mayfly and stonefly richness and abundance were remarkably low, perhaps because of the sluggish nature of streams in the region. With the six new state records, the number of caddisflies known from Indiana has increased to at least 201 species. An updated list is not presented here due to the low number of additions, but the lead author will provide a list upon request.

We do not know how many EPT species reside in INDU, but the fact that 31 species were found at only one of 19 sites strongly suggests that more species will be found. Species estimation at this point is not possible given that the number of singletons (species from a single site or sample) is greater than half of the number of sample units, a prerequisite for using several species richness estimators (Colwell 2013). We would have to double the number of light trap units taken in this study to model richness, a level of sampling not feasible with the resources at hand.

Four sites were comparatively rich in EPT species. A segment of Munson Ditch (Site 3) supported 34 species. Beverly Drive Marsh (Site 4) supported 28 EPT species. The East Arm of the Little Calumet River at Heron Rookery (Site 11) supported 26 species and was the only site to harbor more than one stonefly species. Further investigation of this site is in order, especially since it has yielded some coolwater species such as *Maccaffertium vicarium* (Walker). The East Arm Little Calumet River (Site 16) was also relatively rich with 31 species. Habitats similar to these four will likely yield additional taxa.

DeWalt and South (2015) conducted a similar inventory of EPT on Isle Royale National Park (ISRO) during 2013. They found that the EPT richness of ISRO (73 species) was comparable to INDU, but much lower than found on the mainland surrounding Lake Superior. They also reported that the size of stonefly species inhabiting the island was significantly smaller than that on the mainland—large species being excluded by some factor, presumably the distance (22-70 km from Minnesota or Michigan, respectively) for recolonization after glaciation. Caddisflies again provided over half of the species found, although their diversity was a much smaller proportion of the total EPT richness (57.5% ISRO vs. 82.1% INDU). A shift in family dominance was also evident with Limnephilidae providing the largest percentage of caddisfly richness (21.4%) for ISRO, while at IDNU Leptoceridae was the most species rich family (27.3%). Many of the species recovered from ISRO were cool- or coldwater species with low tolerance for organic enrichment. Conversely, INDU produced mostly warmwater species that were moderately tolerant of organic enrichment and/or low dissolved oxygen levels (see Barbour et al. 1999 for tolerances). Some species of EPT have probably been lost from INDU due to a century of degradation and habitat modification. The lack of a diverse mayfly and stonefly fauna supports this contention.

National Parks and other public properties often protect large proportions of the regional biological community by providing intact habitat and by controlling commercial, industrial, and residential development within their boundaries. Some parks, such as Isle Royale, are isolated, providing considerable protection for communities. Indiana Dunes National Lakeshore is not isolated, its communities are subject to degradation because of the mosaic of public and private property around the park. Nearby there are industries, commerce, and relatively high population densities influencing water and air quality in the park. Still, INDU supports a moderately rich aquatic insect fauna, especially among caddisflies, a fact that would not be known if it were not for inventory work. No reliable baseline assessments for EPT species existed prior to our efforts.

In 2016 the National Park system of the United States will celebrate its 100^th^ anniversary. Next year is also the 50^th^ anniversary of Indiana Dunes National Lakeshore. The authors and volunteers who worked on this project are proud to provide valuable baseline data that will allow for better protection of INDU aquatic systems in the future.

## Supplementary materials

Supplementary material 1 Indiana Dunes EPT Specimen Data Data type: specimen data This dataset is made available under the Open Database License (http://opendatacommons.org/licenses/odbl/1.0/). The Open Database License (ODbL) is a license agreement intended to allow users to freely share, modify, and use this Dataset while maintaining this same freedom for others, provided that the original source and author(s) are credited.R. Edward DeWalt, Eric J. South , Desiree R. Robertson, Joy E. Marburger, Wendy W. Smith, Victoria Brinson
